# Epigenetic modifier Kdm6a/Utx controls the specification of hypothalamic neuronal subtypes in a sex-dependent manner

**DOI:** 10.3389/fcell.2022.937875

**Published:** 2022-10-04

**Authors:** Lucas E. Cabrera Zapata, María Julia Cambiasso, Maria Angeles Arevalo

**Affiliations:** ^1^ Instituto Cajal (IC), CSIC, Madrid, Spain; ^2^ Instituto de Investigación Médica Mercedes y Martín Ferreyra, INIMEC-CONICET, Universidad Nacional de Córdoba, Córdoba, Argentina; ^3^ Facultad de Odontología, Universidad Nacional de Córdoba, Córdoba, Argentina; ^4^ Centro de Investigación Biomédica en Red de Fragilidad y Envejecimiento Saludable (CIBERFES), Instituto de Salud Carlos III, Madrid, Spain

**Keywords:** KDM6A/UTX, H3K27 demethylation, hypothalamic neuronal subtypes, sex differences, neurogenin (Ngn) 3

## Abstract

Kdm6a is an X-chromosome-linked H3K27me2/3 demethylase that promotes chromatin accessibility and gene transcription and is critical for tissue/cell-specific differentiation. Previous results showed higher *Kdm6a* levels in XX than in XY hypothalamic neurons and a female-specific requirement for Kdm6a in mediating increased axogenesis before brain masculinization. Here, we explored the sex-specific role of Kdm6a in the specification of neuronal subtypes in the developing hypothalamus. Hypothalamic neuronal cultures were established from sex-segregated E14 mouse embryos and transfected with siRNAs to knockdown Kdm6a expression (Kdm6a-KD). We evaluated the effect of Kdm6a-KD on Ngn3 expression, a bHLH transcription factor regulating neuronal sub-specification in hypothalamus. Kdm6a-KD decreased Ngn3 expression in females but not in males, abolishing basal sex differences. Then, we analyzed Kdm6a-KD effect on *Ascl1*, *Pomc*, *Npy*, *Sf1*, *Gad1*, and *Th* expression by RT-qPCR. While Kdm6a-KD downregulated *Ascl1* in both sexes equally, we found sex-specific effects for *Pomc*, *Npy*, and *Th*. *Pomc* and *Th* expressed higher in female than in male neurons, and Kdm6a-KD reduced their levels only in females, while *Npy* expressed higher in male than in female neurons, and Kdm6a-KD upregulated its expression only in females. Identical results were found by immunofluorescence for Pomc and Npy neuropeptides. Finally, using ChIP-qPCR, we found higher H3K27me3 levels at *Ngn3*, *Pomc*, and *Npy* promoters in male neurons, in line with Kdm6a higher expression and demethylase activity in females. At all three promoters, Kdm6a-KD induced an enrichment of H3K27me3 only in females. These results indicate that Kdm6a plays a sex-specific role in controlling the expression of transcription factors and neuropeptides critical for the differentiation of hypothalamic neuronal populations regulating food intake and energy homeostasis.

## Introduction

Obesity and its associated comorbidities such as type 2 diabetes mellitus, cardiovascular disease, dyslipidemia, and chronic inflammation, among others, have become a global health threat and a major socioeconomic burden for modern societies with a tendency toward sedentary lifestyles and diets based on hypercaloric/ultra-processed food products (Martin-Rodriguez et al., 2015; Khanna et al., 2022; Lustig et al., 2022). Although it is currently well known that significant sex differences exist in the prevalence, incidence, and severity of a broad range of diseases, including obesity and obesity-related diabetes and cardiovascular disease (Seidell, 2005; Global et al., 2016; Hales et al., 2020; Teufel et al., 2021; Tsao et al., 2022), these differences are not well understood due to a combination of the lack of inclusion of sex as an influencing factor in preclinical studies and the underrepresentation of women in clinical trials. Consequently, there is an urgent international call for future research in the area and a better understanding of sex differences and their mechanistic underpinnings in health and disease (Vasquez-Avila et al., 2021; Regensteiner and Reusch, 2022).

The brain plays a pivotal role in the regulation of energy homeostasis. Particularly, the hypothalamus, one of the most sexually dimorphic brain structures (McEwen et al., 1979; McEwen, 1981; Bao and Swaab, 2010; Pfaff and Christen, 2013; Krause and Ingraham, 2017), is actively involved in the central control of feeding and energy expenditure, sensing and integrating peripheral metabolic and hormonal signals such as glucose, fatty acids, stomach-secreted ghrelin, pancreas-derived insulin, and adipocyte-derived leptin to regulate food intake, glucose metabolism, and energy balance, thereby ensuring that the nutrient demands of the body are fulfilled (Coll and Yeo, 2013; Timper and Bruning, 2017; Uwaifo, 2021). Two functionally antagonistic types of neurons in the arcuate nucleus of the hypothalamus have been identified as major coordinators of these processes: the neuropeptide Y (Npy) and agouti-related peptide (AgRP)-expressing neurons (Npy+ neurons) and the proopiomelanocortin (Pomc)-expressing neurons (Pomc+ neurons; Vohra et al., 2022). Npy+ neurons are orexigenic, meaning they stimulate appetite/hunger and promote feeding behavior (Leibowitz, 1991; Gropp et al., 2005), whereas Pomc+ neurons are anorexigenic, meaning they suppress appetite and promote satiety (Balthasar et al., 2005; Mountjoy, 2015). The appropriate function of these arcuate neuronal circuits and their coordination with other hypothalamic nuclei and extrahypothalamic brain regions also regulating metabolic homeostasis and feeding behavior critically depend on their correct shaping and integration during brain development, the disruption of which, at different levels, can cause a deficient system for the central control of energy balance, often leading to feeding-related diseases such as obesity, type 2 diabetes, and metabolic syndrome, among others (Bingham et al., 2008; Gautron et al., 2015; Timper and Bruning, 2017; Farooqi, 2022). Hypothalamic development during embryogenesis is tightly controlled by basic helix-loop-helix (bHLH) domain-containing transcription factors, which act in a temporally coordinated sequential cascade to determine the neuronal fate and subsequently promote neuronal differentiation (Huang et al., 2014; Bedont et al., 2015; Baker and Brown, 2018). In this cascade of bHLH genes, proneural *Ascl1* (also known as *Mash1*) is expressed first and is required for the subsequent downstream expression of *neurogenin 3* (*Ngn3*; McNay et al., 2006; Pelling et al., 2011; Aujla et al., 2013), which, in turn, regulates the specification of different neuronal subtypes in the hypothalamus, including the Th+ (dopaminergic tyrosine hydroxylase-expressing neurons), Sf1+ (steroidogenic factor 1-expressing neurons), Pomc+, and Npy+ neuronal lineages (Pelling et al., 2011; Anthwal et al., 2013).

Lysine demethylase 6A gene (*Kdm6a*), also known as *Utx*, is located on the X chromosome and encodes a histone demethylase involved in chromatin remodeling. By removing repressive dimethyl and trimethyl (me2/3) groups on lysine (K) at position 27 in histone 3 (H3K27me2/3), Kdm6a promotes chromatin accessibility and allows gene expression (Hong et al., 2007; Tran et al., 2020). In XX individuals, it has been demonstrated in different cell types and developmental stages that *Kdm6a* consistently escapes X chromosome inactivation in both mice and humans and shows transcription from both alleles, leading to higher expression in females (Greenfield et al., 1998; Xu et al., 2008; Armoskus et al., 2014; Berletch et al., 2015; Tukiainen et al., 2017; Davis et al., 2020). Consistent with this, we have previously reported higher levels of *Kdm6a* expression in XX than in XY hypothalamic neurons and a female-specific requirement for the demethylase in mediating increased axogenesis before brain exposure to gonadal hormones (Cabrera Zapata et al., 2021). While Kdm6a is dispensable for the maintenance of embryonic stem cells, it plays a critical role in the determination of neural stem cells and the subsequent differentiation of these pluripotent cells into neurons and glia (Wang et al., 2012; Lei and Jiao, 2018; Yang et al., 2019; Shan et al., 2020; Subhramanyam et al., 2020), with deletion of *Kdm6a* leading to impaired dendritic arborization, synaptic formation, electrophysiological activity and cognition (Tang et al., 2017; Tang et al., 2020), and loss-of-function mutations in *KDM6A* causing cognitive deficits in humans (Miyake et al., 2013; Van Laarhoven et al., 2015; Bogershausen et al., 2016; Faundes et al., 2021). Herein, we investigated the role of Kdm6a in the specification of neuronal subtypes in the developing hypothalamus and its differential requirements in males and females, focusing mainly on Pomc+ and Npy+ neuronal populations as essential elements in the central control of food intake and energy homeostasis.

## Materials and methods

### Animals

CD1 mice raised in our in-house colony at the Instituto Cajal (CSIC, Madrid, Spain) were used for this study. Animals received water and food *ad libitum* and were kept in controlled macroenvironmental conditions of temperature at 22 ± 2°C and a 12 h light/12 h dark periodic cycle. Procedures for care, welfare, and proper use of all experimental animals followed the European Parliament and Council Directive (2010/63/EU) and the Spanish regulation (R.D. 53/2013 and Ley 6/2013, 11th June) and were approved by our Institutional Animal Care and Use Committee (Comité de Ética de Experimentación Animal del Instituto Cajal) and by the Consejería del Medio Ambiente y Territorio (Comunidad de Madrid, PROEX 134/17).

### Hypothalamic neuronal cultures

CD1 mouse embryos at 14 days of gestation (E14, defining E0 as the day of the vaginal plug) were used to establish primary hypothalamic neuronal cultures. Donor embryo age was specifically selected with the purpose of avoiding exposure of neurons to the peak in gonadal testosterone secretion during *in utero* development, which occurs in male mice around E17 (O'Shaughnessy et al., 2006). Pregnant females were sacrificed by cervical dislocation under CO_2_ anesthesia, and embryos were dissected from the uterus. Neurons were cultured separately according to sex by observing the presence/absence of the spermatic artery in the developing gonads of embryos. The ventromedial hypothalamic region was dissected out and stripped off the meninges. Blocks of tissue were incubated for 15 min at 37°C with 0.5% trypsin (Gibco, United States) and then washed three times with Ca^2+^/Mg^2+^-free Hank’s buffered salt solution (Gibco). Finally, tissue was mechanically dissociated into single cells in a 37 °C warm culture medium, and cells were seeded. The medium was phenol red-free Neurobasal (Gibco) to avoid “estrogen-like effects” (Berthois et al., 1986) and was supplemented with B-27, 0.043% L-alanyl-l-glutamine (GlutaMAX-I) and 1% antibiotic-antimycotic containing 10.000 U/ml penicillin, 10.000 μg/ml streptomycin, and 25 μg/ml amphotericin B (Gibco). Cells were plated on 6-well plates (Falcon, United States ) at a density of 500–1000 cells/mm^2^ or on 10 mm glass coverslips (Assistent, Germany) at a density of 800 cells/mm^2^ for RT-qPCR/ChIP-qPCR or immunofluorescence, respectively. The surfaces of glass coverslips and plates were pre-coated with 1 μg/μL poly-l-lysine (Sigma-Aldrich, United States).

### Small interfering RNA (siRNA) transfection

Neurons were transfected by electroporation or lipofection using a mixture of four different siRNA sequences targeting *Kdm6a* transcripts at a final concentration of 40 nM total RNA (ON-TARGETplus Mouse Kdm6a set of four siRNA, Dharmacon, UK); procedures and knockdown efficacy were previously described and demonstrated (Cabrera Zapata et al., 2021). A non-targeting siRNA sequence (ntRNA; Dharmacon) was used as a control, and co-transfection with pmaxGFP (Lonza, Switzerland) was performed in all cases for transfected neuron identification. For immunofluorescence, neurons were transfected by lipofection at 3 days *in vitro* (DIV) with target siRNA or ntRNA using Effectene Transfection Reagent (Qiagen, Germany) according to the manufacturer’s instructions, and after 18 h of knockdown, they were fixed and immunolabeled. For gene expression analysis and ChIP assays, neurons were transfected by electroporation before seeding with target siRNA or ntRNA using a 4D-Nucleofector X Unit and the corresponding P3 Primary Cell nucleofection kit (Lonza) according to the manufacturer’s instructions, seeded, and incubated for three DIV until processing for RNA isolation/ChIP reactions.

### RNA isolation and reverse transcription quantitative real-time PCR (RT-qPCR)

Total RNA was extracted using TRIzol reagent (Invitrogen, United States), purified, and quantified by spectrophotometry on NanoDrop One (Thermo Fisher Scientific, United States) as previously described (Cisternas et al., 2015). Then, 1 µg of RNA per sample was reverse transcribed to cDNA in a 20 µL reaction using M-MLV reverse transcriptase (Promega, United States) and random primers (Invitrogen), following the manufacturer’s instructions. qPCR reactions were performed on a 7500 real-time PCR system (Applied Biosystems, United States) using TaqMan or SYBR Green Universal PCR Master Mix (Applied Biosystems). TaqMan probes and primers for *Ngn3* were assay-on-demand gene expression products (Applied Biosystems). All other primers (Table 1) were designed using the online primer-basic local alignment search tool (Primer-BLAST; National Institutes of Health, United States), selecting primer pairs spanning an exon–exon junction to restrict amplification specifically to mRNA. All primers were verified to amplify with a 95–100% efficiency by performing 4-point calibration curves. Relative quantification of mRNA expression was determined with the ΔΔCt method, using the BestKeeper index (Pfaffl et al., 2004) calculated for each sample from the Ct values of *Rn18s* (18S rRNA) and *Rpl13a* as control housekeeping genes. Control male samples were used as a reference group.

**TABLE 1:** Primer sequences used for qPCR assays.

*Gene*	Forward sequence 5′‒3′ Reverse sequence 5′‒3′
*Ascl1*	ACT​TTG​GAA​GCA​GGA​TGG​CAG
	TTA​GTG​AAG​GTG​CCC​CTG​TAG
*Ascl1* promoter	GTG​TCC​CAT​TGA​AAA​GGC​GG
	AAT​TGC​TCT​CTC​GTT​CCC​CC
*Gad1*	GTCGCTGAACCGAGCCTG
	GTG​GTC​TTG​GGG​TCT​CTA​CG
*Ngn3* promoter	GCA​GAG​CAG​ATA​AAG​CGT​GC
	TCG​CCT​GGA​GTA​AAT​TGC​GT
*Npy*	CCA​TGT​GGT​GAT​GGG​AAA​TG
	ATT​GGT​GGG​ACA​GGC​AGA​CT
*Npy* promoter	CGACAAGGGCGCTCCATA
	GCC​TCT​GTG​AGA​GAA​GAG​ATC​C
*Pomc*	AGG​TGT​ACC​CCA​ACG​TTG​CT
	GAC​CTG​CTC​CAA​GCC​TAA​TGG
*Pomc* promoter	CCA​GGA​AGG​TCA​CGT​CCA​AG
	GTT​TGG​TCC​CTG​TCG​CTC​TT
*Rn18s*	CGC​CGC​TAG​AGG​TGA​AAT​TCT
	CAT​TCT​TGG​CAA​ATG​CTT​TCG
*Rpl13a*	TAC​CAG​AAA​GTT​TGC​TTA​CCT​GGG
	TGC​CTG​TTT​CCG​TAA​CCT​CAA​G
*Sf1*	GCG​GGC​ATG​GAC​TAT​TCG​TA
	CTT​GAA​GAA​GCC​CTT​GCA​GC
*Th*	AGG​GCC​TCT​ATG​CTA​CCC​AT
	AAG​CCA​GTC​CGT​TCC​TTC​AA

### Immunofluorescence

Neurons were fixed for 20 min at room temperature (RT) in 4% paraformaldehyde prewarmed to 37°C, rinsed, permeabilized for 10 min with 0.5% Triton X-100 (Bio-Rad, United States ) in phosphate-buffered saline (PBS), blocked 1 h at RT in 1% BSA-PBS solution, and incubated for 1 h at RT with the following antibodies diluted 1:1000 in 1% BSA-PBS: anti-Ngn3 mouse monoclonal antibody (F25A1B3, deposited by Madsen, O.D. to the Developmental Studies Hybridoma Bank, NICHD-NIH, maintained at The University of Iowa, Department of Biology, Iowa City, United States ), anti-Pomc rabbit polyclonal antibody (H-029–30, Phoenix Pharmaceuticals, United States ), or anti-Npy rabbit polyclonal antibody (T-4070, Peninsula Laboratories-BMA Biomedicals, Switzerland). After rinsing with PBS, cells were incubated for 1 h at RT with the secondary antibodies HRP goat anti-mouse (1:200 in 1% BSA-PBS) followed by a 10-min amplification reaction with tyramide for the detection of Ngn3 (Tyramide Amplification Kit, 33,003, Biotium, United States ), or Alexa 594 donkey anti-rabbit for the detection of Pomc/Npy (1:1000 in 1% BSA-PBS; Jackson ImmunoResearch, United States ). Finally, neurons were mounted on glass slides using gerbatol (0.3 g/ml glycerol, 0.13 g/ml Mowiol, 0.2 M Tris-HCl, pH 8.5) plus 1:5000 DAPI for nuclei staining.

### Imaging and quantitative image analysis

Imaging was carried out at ×40 magnification using a standard Leica DMI 6000 fluorescence microscope (Leica, Germany) equipped with a digital camera of the same firm. To quantify fluorescent intensity, the soma of GFP-expressing neurons was outlined, and the area and integrated density were measured in the corresponding channel for Ngn3, Pomc, or Npy immunofluorescence signal using Fiji-ImageJ software (NIH, United States ; freely available at https://imagej.nih.gov/ij/). Background fluorescence was also measured for each neuron and image analyzed. From these values, corrected total cell fluorescence (CTCF) intensity was calculated using the following equation (Martin Fitzpatrick, University of Birmingham, UK, available at https://theolb.readthedocs.io/en/latest/):

CTCF=integrated density of soma−(area of soma×background fluorescence).

Thus, 20–40 GFP-expressing (GFP+) neurons were randomly measured per experimental condition and culture (four independent cultures). An equivalent number of non-GFP-expressing (GFP-) neurons transfected with targeting siRNA were randomly measured per culture to confirm that changes in CTCF were not a methodological artifact.

### Chromatin immunoprecipitation (ChIP) analysis

ChIP assays were performed with the ChIP-IT High Sensitivity Kit (Active Motif, United States ) strictly following the manufacturer’s instructions. Briefly, ∼7–8 million hypothalamic neurons segregated by sex were cultured during three DIV and then fixed for 15 min with a fixation buffer containing 1.1% formaldehyde (Sigma-Aldrich). Cross-linked cells were scraped, centrifuged, washed three times with cold PBS, and resuspended in ChIP buffer supplemented with PIC (Protease Inhibitor Cocktail; Active Motif) and PMSF (phenylmethylsulfonyl fluoride; Active Motif). Next, cells were homogenized using a Dounce homogenizer with a tight-fitting pestle, and the chromatin was sheared to ∼400–800 bp fragments by sonication with a Fisher Scientific Model 705 Sonic Dismembrator ultrasonic processor equipped with a microtip for small volume samples (FB705220, Thermo Fisher Scientific; 30 cycles 30″ ON/30″ OFF at 4°C). Then, 1% of the total sonicated chromatin was kept as the input DNA and also used to determine the DNA concentration at 260 nm on NanoDrop One (Thermo Fisher Scientific) for each sample. ChIP reactions were carried out overnight at 4 °C with 3 µg of sheared chromatin and 10 μg of anti-H3K27me3 rabbit polyclonal antibody (39155, Active Motif) or anti-mouse IgG as negative control (115-001-003, Jackson ImmunoResearch) in ChIP buffer supplemented with PIC. Reactions were then incubated for 3 h with pre-cleared protein G agarose beads on an end-to-end rotator at 4°C. Then, samples were filtered using the kit columns, washed five times while remaining on columns with wash buffer AM1, and centrifuged to elute the ChIP DNA. Reversal of cross-links was performed by incubating the samples with proteinase K in a thermocycler at 55°C for 30 min plus 2 h at 80°C. Finally, DNA was purified using purification columns and buffers supplied in the kit. Immunoprecipitated DNA was quantified by real-time qPCR using primer pairs for the promoter regions of *Ascl1, Ngn3*, *Pomc*, and *Npy* (Table 1) and the SYBR Green Master Mix and device detailed earlier. ChIP-qPCR data were normalized to the input DNA and expressed as a percent of the input.

### Statistical analysis

Data are presented as mean ± SEM and were statistically evaluated by two-way analysis of variance (ANOVA) with treatment/ChIP and gonadal sex as independent variables. The statistical significance of the effects of each independent variable and their interactions was tested. *Posthoc* comparisons of means by Fisher’s least significant difference (LSD) test were performed for those variables/interactions for which ANOVA *p*-values were statistically significant. Statistical analysis was performed entirely with Statistica 8 software (StatSoft Inc., United States ). P < 0.05 was considered statistically significant. Sample size (n) is indicated in the figure legends and was 3–6 independent cultures for RT-qPCR/ChIP-qPCR experiments or 40–100 transfected neurons from at least four independent cultures for immunofluorescence analysis. The number of independent cultures corresponds to the number of pregnant mothers from which embryos were obtained.

## Results

### Kdm6a is female-specifically required for the expression of higher levels of Ngn3 in female than in male hypothalamic neurons

**FIGURE 1:**
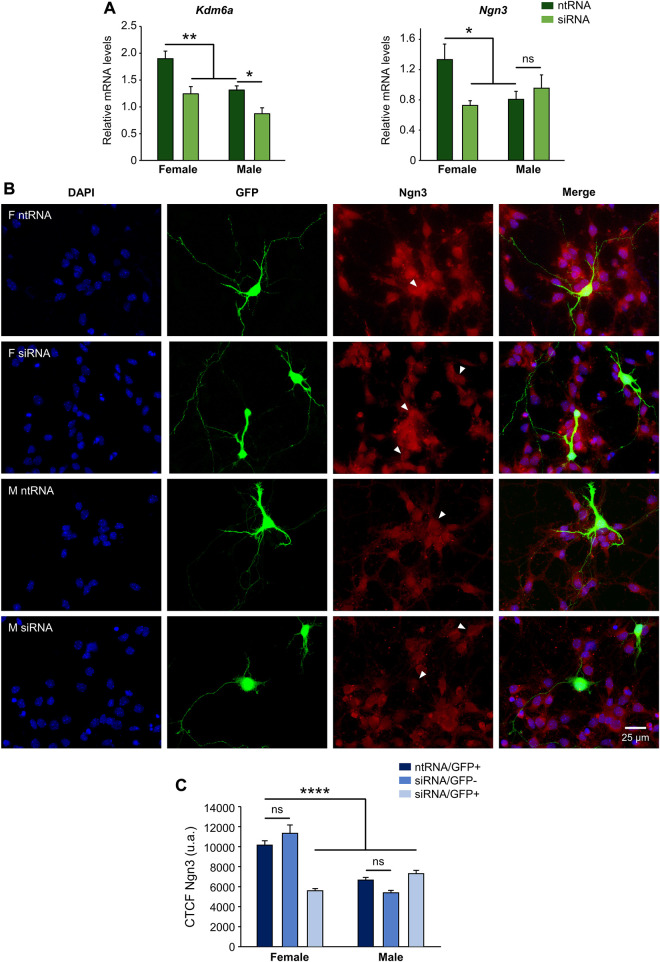
Kdm6a is required for the sexually dimorphic expression of proneural Ngn3. **(A)** Kdm6a knockdown effect on *Kdm6a* and *Ngn3* gene expression analyzed by RT-qPCR in male- and female-derived hypothalamic neurons transfected with a control non-targeting siRNA sequence (ntRNA) or siRNA targeting Kdm6a (siRNA). *Kdm6a* and *Ngn3* showed higher expression levels in female than in male neurons under control conditions. siRNA targeting Kdm6a was effective in downregulating the demethylase mRNA levels in both male and female neurons. Kdm6a knockdown by siRNA decreased *Ngn3* mRNA levels only in female neurons. **(B)** Representative fluorescence images of female (F) and male (M) hypothalamic neurons co-transfected with ntRNA and GFP or siRNA and GFP at three DIV for 18 h. Ngn3 protein expression (red) was determined by immunofluorescence staining in transfected GFP+ neurons (green). Nuclei were stained with DAPI (blue). Arrowheads point to representative measured neuronal somas. **(C)** Quantification of fluorescence intensity for Ngn3 expressed as corrected total cell fluorescence (CTCF). Kdm6a knockdown by siRNA eliminated sex differences in Ngn3 protein expression by downregulating the proneural factor only in female-derived neurons. Data are mean ± SEM. *n* = 40–100 neurons from four independent cultures for each sex and treatment. ns, not significant; *p < 0.05; **p < 0.01; ****p < 0.0001.

We have previously reported higher levels of expression of *Kdm6a* in hypothalamic neurons carrying two X chromosomes compared to those carrying one X and one Y chromosome regardless of gonadal sex, as well as a requirement for this higher *Kdm6a* expression and H3K27 demethylation activity in females for the increased expression of *Ngn3* in females compared to male hypothalamic neurons at a transcriptional (mRNA) level (Cabrera Zapata et al., 2021). Herein, we first proved the effectiveness of siRNAs designed to knockdown *Kdm6a* expression by transfecting hypothalamic neuronal cultures derived from sex-segregated E14 mice and measuring the effect on Kdm6a mRNA levels (Figure 1A; two-way ANOVA: F (1, 19) = 18.12, *p* = 0.0004). In addition, as previously described (Cabrera Zapata et al., 2021), we confirmed the effect of Kdm6a knockdown on decreasing *Ngn3* mRNA levels in females but not in male neurons (Figure 1A; two-way ANOVA, sex-treatment interaction effect: F (1, 10) = 6.169, *p* = 0.032). Finally, to determine whether Kdm6a is also required for the sexually dimorphic expression of Ngn3 at the protein level, hypothalamic neuronal cultures were transfected with the Kdm6a-targeting siRNAs, and the effect of Kdm6a knockdown on Ngn3 protein expression was analyzed by immunofluorescence. Remarkably, consistent with results evaluating mRNA levels, Kdm6a silencing led to a significant decrease in Ngn3 protein levels only in female-derived neurons without affecting male cultures, abolishing the sex differences for Ngn3 expression observed under control conditions (Figures 1B and C; two-way ANOVA, sex-treatment interaction effect: F (2, 358) = 32.18, *p* < 0.0000001).

### Kdm6a is required for Ascl1 transcription and regulates the expression of Th, Pomc, and Npy in a sexually dimorphic manner

**FIGURE 2:**
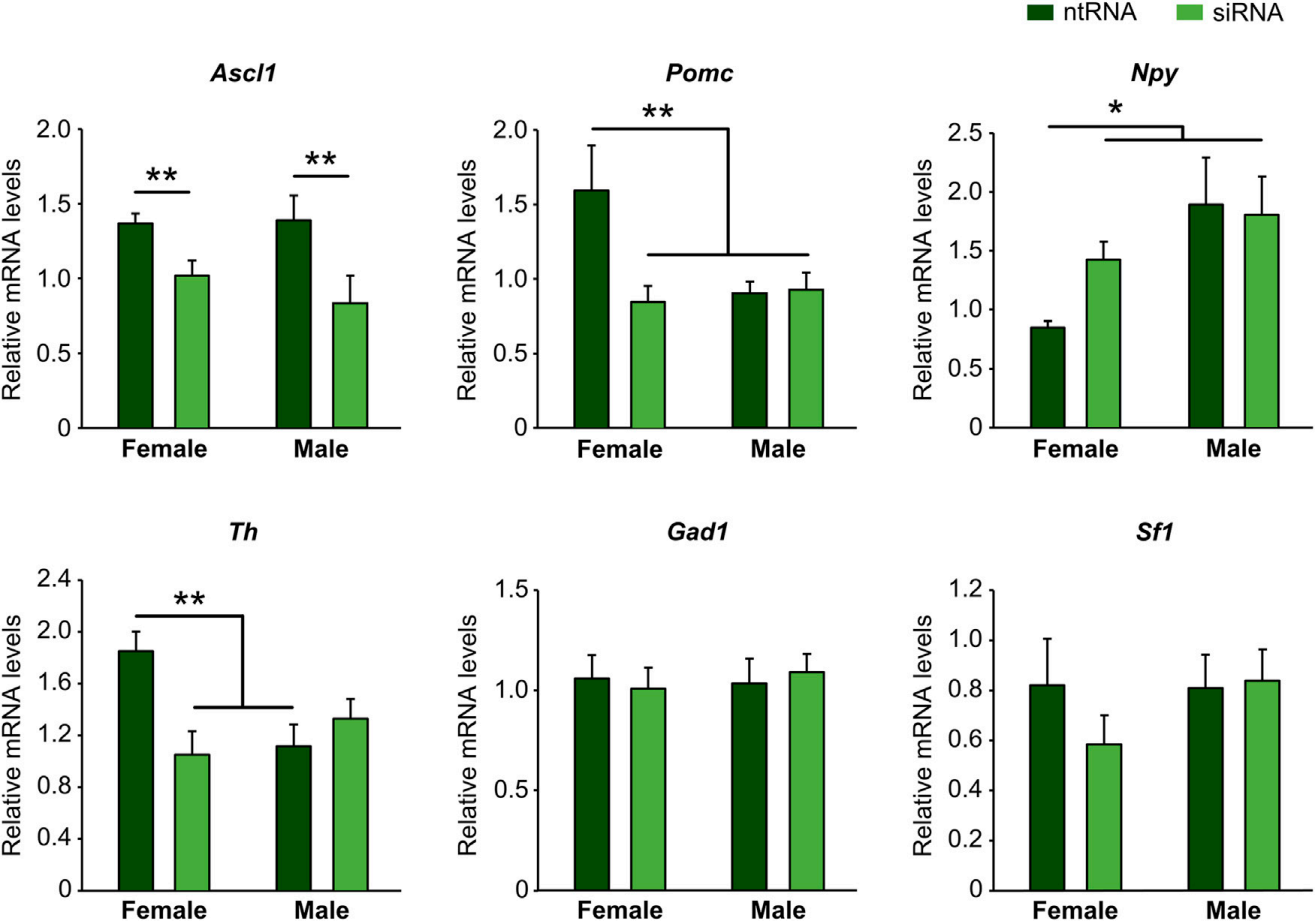
Kdm6a regulates gene expression of molecular markers of different hypothalamic neuronal lineages in a sex-specific manner. Kdm6a knockdown effect on *Ascl1*, *Pomc*, *Npy*, *Th*, *Gad1*, and *Sf1* gene expression analyzed by RT-qPCR in male- and female-derived hypothalamic neurons transfected with a control non-targeting siRNA sequence (ntRNA) or siRNA targeting Kdm6a (siRNA). Data are mean ± SEM. n = 4–6 independent cultures for each sex and treatment. *p < 0.05; **p < 0.01.

Ngn3 has been shown to play an essential role in the specification of different neuronal subtypes in the hypothalamus, having opposing effects such as promotion of Pomc+ and Sf1+ and repression of Npy+ and Th+ fate (Pelling et al., 2011; Anthwal et al., 2013). However, such effects have not been properly addressed by considering sex as a crucial factor in hypothalamus development. Since we have demonstrated that Kdm6a has a sexually dimorphic expression pattern itself and that it is specifically required in female-derived hypothalamic neurons to upregulate Ngn3 expression, then we analyzed by RT-qPCR the effect of Kdm6a downregulation by siRNAs on mRNA expression levels of *Ascl1*, *Pomc*, *Npy*, *Th*, *Gad1*, and *Sf1*, all of them being molecular markers of different hypothalamic neuronal lineages (Romanov et al., 2020). Whereas Kdm6a knockdown downregulated mRNA levels of the transcription factor *Ascl1* in both sexes equally (Figure 2; two-way ANOVA, treatment main effect: F (1, 15) = 10.05, *p* = 0.006), sex-specific effects were found for *Pomc*, *Npy*, and *Th*. Interestingly, *Pomc* and *Th* were significantly more expressed in neurons derived from females than from males, and Kdm6a silencing reduced their mRNA levels only in females (Figure 2; two-way ANOVA, sex-treatment interaction effect: Pomc: F (1, 14) = 5.76, *p* = 0.031; Th: F (1, 12) = 9.625, *p* = 0.009), while *Npy* expression was higher in male than in female-derived neurons in control conditions, and Kdm6a knockdown upregulated its expression only in females (Figure 2; two-way ANOVA, sex-treatment interaction effect: F (1, 13) = 6.46, *p* = 0.025). No effects of sex or Kdm6a knockdown on *Sf1* or *Gad1* mRNA levels were found (Figure 2).

**FIGURE 3:**
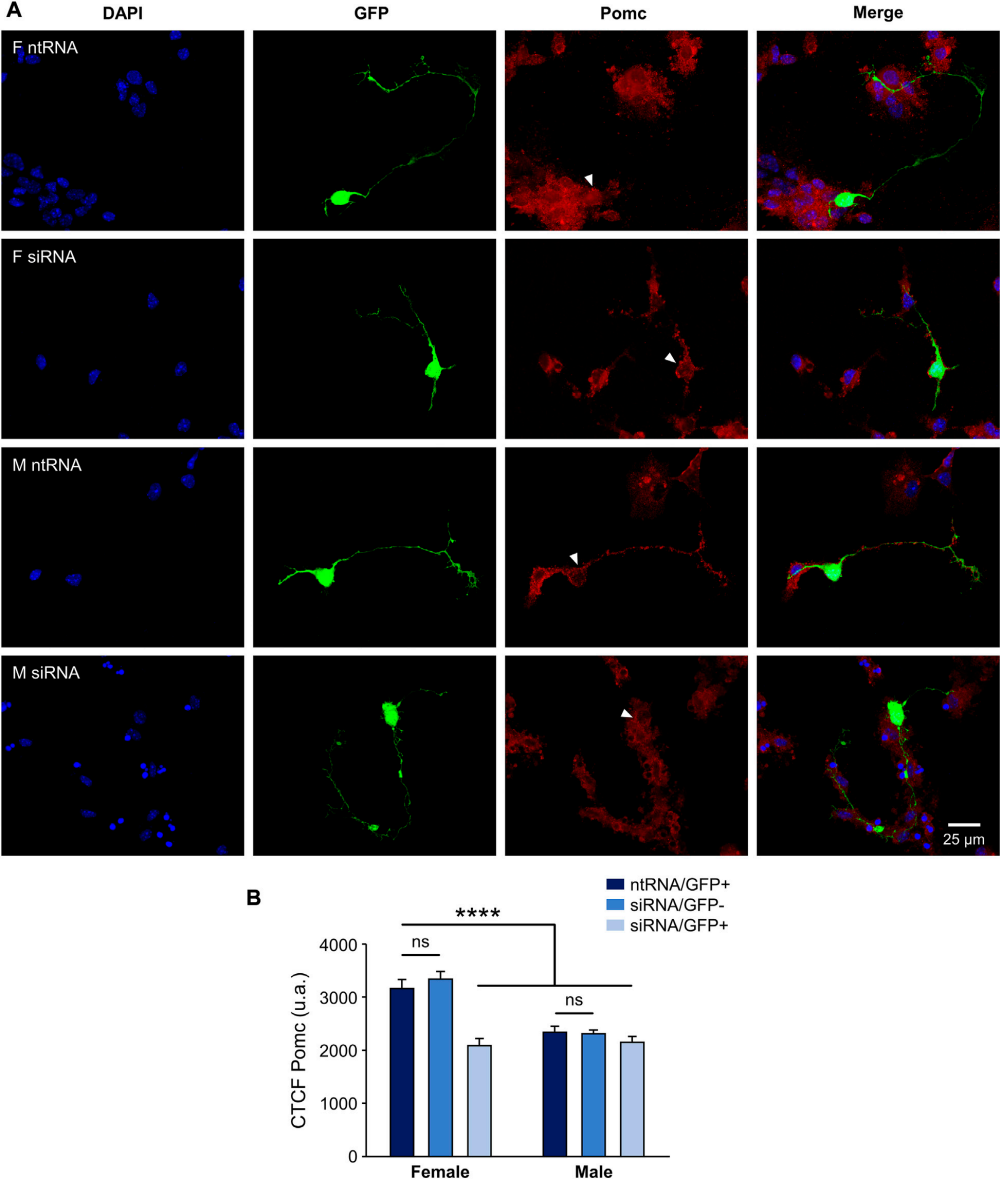
Kdm6a is required for Pomc sexually dimorphic expression. **(A)** Representative fluorescence images of female (F) and male (M) hypothalamic neurons co-transfected with a non-targeting siRNA sequence (ntRNA) and GFP or siRNA targeting Kdm6a (siRNA) and GFP at three DIV for 18 h. Pomc protein expression (red) was determined by immunofluorescence staining in transfected GFP+ neurons (green). Nuclei were stained with DAPI (blue). Arrowheads point to representative measured neuronal somas. **(B)** Quantification of fluorescence intensity for Pomc expressed as corrected total cell fluorescence (CTCF). Kdm6a knockdown by siRNA abolished sex differences in Pomc protein expression by downregulating the neuropeptide only in female-derived neurons. Data are mean ± SEM. n = 55–80 neurons from four independent cultures for each sex and treatment. ns, not significant; ****p < 0.0001.

**FIGURE 4:**
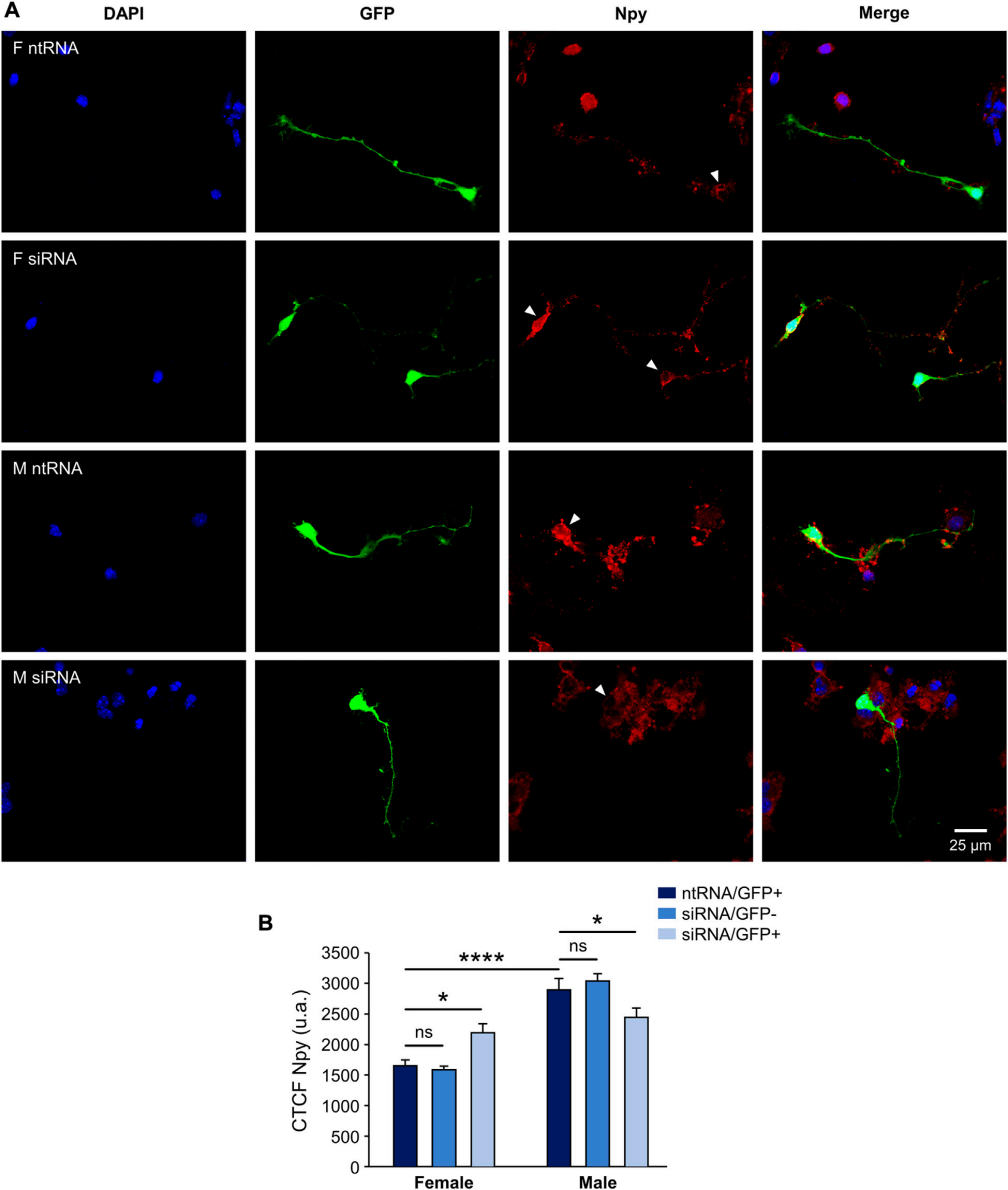
Kdm6a is required for Npy sexually dimorphic expression. **(A)** Representative fluorescence images of female (F) and male (M) hypothalamic neurons co-transfected with a non-targeting siRNA sequence (ntRNA) and GFP or siRNA targeting Kdm6a (siRNA) and GFP at three DIV for 18 h. Npy protein expression (red) was determined by immunofluorescence staining in transfected GFP+ neurons (green). Nuclei were stained with DAPI (blue). Arrowheads point to representative measured neuronal somas. **(B)** Quantification of fluorescence intensity for Npy expressed as corrected total cell fluorescence (CTCF). Npy expressed higher in male than in female hypothalamic neurons, and Kdm6a knockdown by siRNA increased the neuropeptide expression in females while decreasing it in males. Data are mean ± SEM. *n* = 60–90 neurons from four independent cultures for each sex and treatment. ns, not significant; *p < 0.05; ****p < 0.0001.

Having found sexually dimorphic mRNA expression patterns for *Pomc* and *Npy* regulated by Kdm6a in a sex-specific way, and considering the opposing roles of Pomc+ and Npy+ hypothalamic neurons in regulating food intake and energy balance in mammals (Morton et al., 2006; Chen et al., 2022), we decided to analyze the effect of Kdm6a silencing on Pomc and Npy protein expression levels by immunolabeling neurons co-transfected with siRNAs and pmaxGFP. Fluorescence intensity analysis for Pomc and Npy proteins in GFP-expressing hypothalamic neurons showed results consistent with the previous assessment at the mRNA level. As shown in Figure 3, Pomc protein expression was significantly decreased after Kdm6a knockdown in female but not in male-derived hypothalamic neurons, erasing the sex differences in the expression levels of this neuropeptide observed in control conditions (two-way ANOVA, sex-treatment interaction effect: F (2, 361) = 9.52, *p* = 0.0001). Regarding Npy, male hypothalamic neurons expressed significantly higher levels of this neuropeptide than female neurons under control conditions, whereas Kdm6a silencing had opposite effects in each sex, leading to increased Npy expression in females and decreased expression in males (Figure 4; two-way ANOVA, sex-treatment interaction effect: F (2, 365) = 8.2, *p* = 0.0003).

### Kdm6a actively controls H3K27me3 demethylation at *Ngn3*, *Pomc*, and *Npy* promoters in female hypothalamic neurons

**FIGURE 5:**
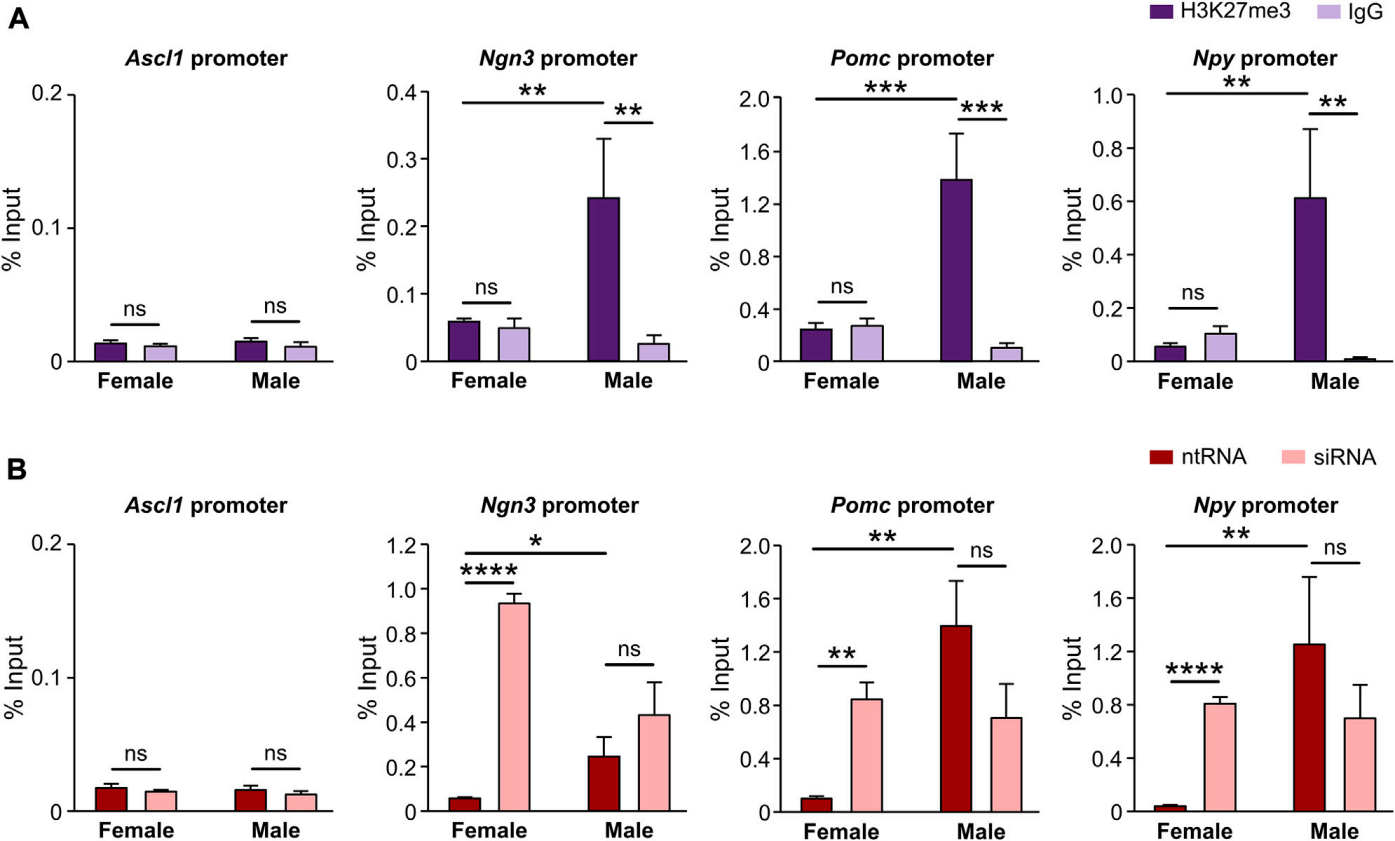
Promoters of *Ngn3*, *Pomc*, and *Npy* but not *Ascl1* are specifically regulated by Kdm6a H3K27me3 demethylase activity. **(A)** Levels of *Ascl1*, *Ngn3*, *Pomc*, and *Npy* promoters DNA bound by H3K27me3 in control hypothalamic neurons measured by ChIP-qPCR and expressed as % of input DNA. IgG antibody served as a negative control for H3K27me3 immunoprecipitation. **(B)** Kdm6a knockdown effect on *Ascl1*, *Ngn3*, *Pomc*, and *Npy* promoters DNA bound by H3K27me3 in male- and female-derived hypothalamic neurons transfected with a control non-targeting siRNA sequence (ntRNA) or siRNA targeting Kdm6a (siRNA). Data are mean ± SEM. n = 3–4 independent cultures for each group. ns, not significant; *p < 0.05; **p < 0.01; ***p < 0.001; ****p < 0.0001.

Given the results clearly demonstrating that Kdm6a regulates Ascl1, Ngn3, Pomc, and Npy expression in hypothalamic neurons in a sex-dependent manner in all cases except for Ascl1, and knowing that Kdm6a promotes gene transcription mainly by demethylating H3K27me3, we performed ChIP-qPCR assays in sex-segregated neuronal cultures to evaluate whether the promoter regions of *Ascl1, Ngn3*, *Pomc*, and *Npy* genes are being controlled by the H3K27me3 histone epigenetic modification. Results expressed as qPCR data for promoter sequences in H3K27me3 or IgG (negative control) ChIP samples normalized to input (starting non-immunoprecipitated chromatin) confirmed the presence of the repressive H3K27me3 mark removed by Kdm6a at the promoters of all analyzed genes except *Ascl1*, showing that the expression of *Ngn3*, *Pomc*, and *Npy* is regulated by H3K27 methylation/demethylation. Notably, in all positive cases, we found significantly higher levels of promoter DNA bound to H3K27me3 in male than in female neurons, indicating a more suppressive effect for *Ngn3*, *Pomc*, and *Npy* transcription in males (Figure 5A; two-way ANOVA, sex-ChIP interaction effect: *Ngn3*: F (1, 9) = 6.29, *p* = 0.03; *Pomc*: F (1, 9) = 15.41, *p* = 0.003; *Npy*: F (1, 9) = 7.34, *p* = 0.02). Finally, Kdm6a silencing led to a significant increase in H3K27 trimethylation at these promoters in female but not in male neurons (Figure 5B; two-way ANOVA, sex-treatment interaction effect: *Ngn3*: F (1, 9) = 17.85, *p* = 0.002; *Pomc*: F (1, 11) = 9.25, *p* = 0.011; *Npy*: F (1, 10) = 6.5, *p* = 0.029). These results are in line with the higher expression of Kdm6a and H3K27 demethylase activity in female than in male hypothalamic neurons as previously reported (Cabrera Zapata et al., 2021).

## Discussion

In this work, we present novel data suggesting a sex-specific role for the histone demethylase Kdm6a in the sexually dimorphic differentiation and specification of neuronal subtypes in the hypothalamus. Kdm6a was female-specifically required for the expression of higher levels of the proneural transcription factor Ngn3 in female than in male hypothalamic neurons. Importantly, we have shown a Kdm6a sex-specific requirement for Pomc, Npy, and Th sexually dimorphic expression in hypothalamic neurons. ChIP-qPCR data confirmed the presence of H3K27me3 repressive epigenetic marks regulating chromatin accessibility at *Ngn3*, *Pomc*, and *Npy* promoter regions, indicating a role for Kdm6 demethylases in controlling transcription of these genes by removing H3K27 methylation. Notably, in all three genes, we found significantly higher levels of promoter DNA bound to H3K27me3 in male than in female neurons, indicating a more suppressive effect for their transcription in males. Finally, Kdm6a silencing induced a significant enrichment of H3K27 trimethylation at the promoters of these genes only in female neurons, showing a specific requirement of Kdm6a for demethylation and chromatin accessibility at these loci in females. All these results clearly reveal dimorphic mechanisms for hypothalamic neuronal differentiation that depend on the X-linked epigenetic regulator Kdm6a and arise early in embryogenesis and before the critical action of gonadal hormones in the brain, thus contributing to the understanding of the role of sex chromosomes in brain sexual differentiation beyond the effects of gonadal hormones.

The bHLH transcription factor Ngn3 plays an essential role in neuronal development and diversification of neuronal subtypes in the hypothalamus. Performing genetic fate mapping studies and using *Ngn3*-null mutant mice (Gradwohl et al., 2000), it was possible to demonstrate that Ngn3+ progenitors contribute to subsets of arcuate Th+, Pomc+, and Npy+ neurons and ventromedial Sf1+ neurons. Although the requirement for Ngn3 was different depending on the neuronal subtype, while Ngn3 promoted the development of Pomc+ and Sf1+ neurons, it inhibited the development of Npy+ and Th+ neurons (Pelling et al., 2011). Moreover, conditional deletion of *Ngn3* specifically in the developing embryonic hypothalamus by using a *Nkx2.1iCre* transgenic mouse led in adulthood to a depletion of *Pomc* expression in arcuate neurons, a decrease in leptin sensitivity in ventral hypothalamic areas, and an associated obesity due to hyperphagia and reduced energy expenditure (Anthwal et al., 2013). However, although differences between male and female *Ngn3* conditional-knockout mice in parameters such as body weight gain and insulin and leptin insensitivity were reported (with more pronounced effects in females than in males; Anthwal et al., 2013), sex differences regarding Ngn3 role in the specification of hypothalamic neuronal subtypes have not been properly addressed. The hypothalamus is a brain region well known to be a highly sexually dimorphic structure (McEwen et al., 1979; McEwen, 1981; Pfaff and Christen, 2013), and our groups have already reported higher *Ngn3* expression levels in female than in male primary hypothalamic (Scerbo et al., 2014; Cisternas et al., 2020) and hippocampal (Ruiz-Palmero et al., 2016) neurons. In the present study, Kdm6a knockdown downregulated Ngn3 mRNA and protein expression only in female hypothalamic neurons without affecting it in male neurons, clearly indicating a female-specific requirement of Kdm6a for higher Ngn3 levels in female than in male neurons. Furthermore, Kdm6a silencing affected the transcription of recognized molecular markers of different hypothalamic neuronal lineages, such as *Ascl1*, *Th*, *Pomc*, and *Npy*. *Ascl1* (formerly known as *Mash1*) is another proneural bHLH transcription factor controlling both neurogenesis and neuronal subtype specification in the hypothalamus (McNay et al., 2006; Alvarez-Bolado, 2019; Romanov et al., 2020), which has been reported to initiate a cascade of bHLH genes during early embryogenesis by inducing *Ngn3* expression that, in turn, promotes *Neurod1* and *Nhlh2* transcription downstream (McNay et al., 2006; Pelling et al., 2011). Our data show a requirement of Kdm6a for *Ascl1* mRNA expression equally important in male and female hypothalamic neurons, suggesting a key regulatory role for Kdm6a in neurogenesis and neuronal diversification in the hypothalamus through the transcriptional control of a proneural master gene such as *Ascl1*. These results are not only relevant considering the proneural functions of Ascl1 during hypothalamic development *in utero* but could also be of critical interest in the exciting, more recent, and rapidly developing field of postnatal-adult hypothalamic neurogenesis (Kokoeva et al., 2005, 2007; Hourai and Miyata, 2013; Yoo and Blackshaw, 2018), where Ascl1 expression has been proposed to regulate the switch from the quiescent to the active state of neural stem cells (Sueda et al., 2019; Dou et al., 2021; Son et al., 2021). However, ChIP-qPCR assays found neither H3K27me3 enrichment nor any change in H3K27me3 levels after Kdm6a knockdown at the *Ascl1* promoter, indicating that Kdm6a-mediated mechanisms other than histone demethylation are acting to promote *Ascl1* transcription in both male and female hypothalamic neurons. This is not surprising, as histone demethylation-independent functions for Kdm6a in the regulation of chromatin accessibility and gene transcription have been reported (Shpargel et al., 2012; Wang et al., 2012; Wang et al., 2017). Further studies including ChIP analysis directly targeting Kdm6a and co-immunoprecipitation assays should be performed to assess Kdm6a occupancy at the *Ascl1* locus and to identify possible molecular co-regulators with which Kdm6a may be interacting to promote *Ascl1* transcription. Additionally, we found higher mRNA levels of the dopaminergic molecular marker *Th* in female than in male-derived cultures and a female-specific requirement of Kdm6a for the expression of this sexually dimorphic pattern. Remarkably, it has recently been demonstrated that all Th+ dopaminergic neurons in the hypothalamus are derived from Ascl1-expressing progenitors (Romanov et al., 2020) and that hypothalamic dopaminergic fate is modulated through the Ascl1/Ngn3 pathway (McNay et al., 2006; Pelling et al., 2011). Herein, we have shown a transcriptional regulatory role for Kdm6a at the three different levels of the Ascl1/Ngn3/Th axis, with sex-specific effects at Ngn3 and Th levels.

**FIGURE 6:**
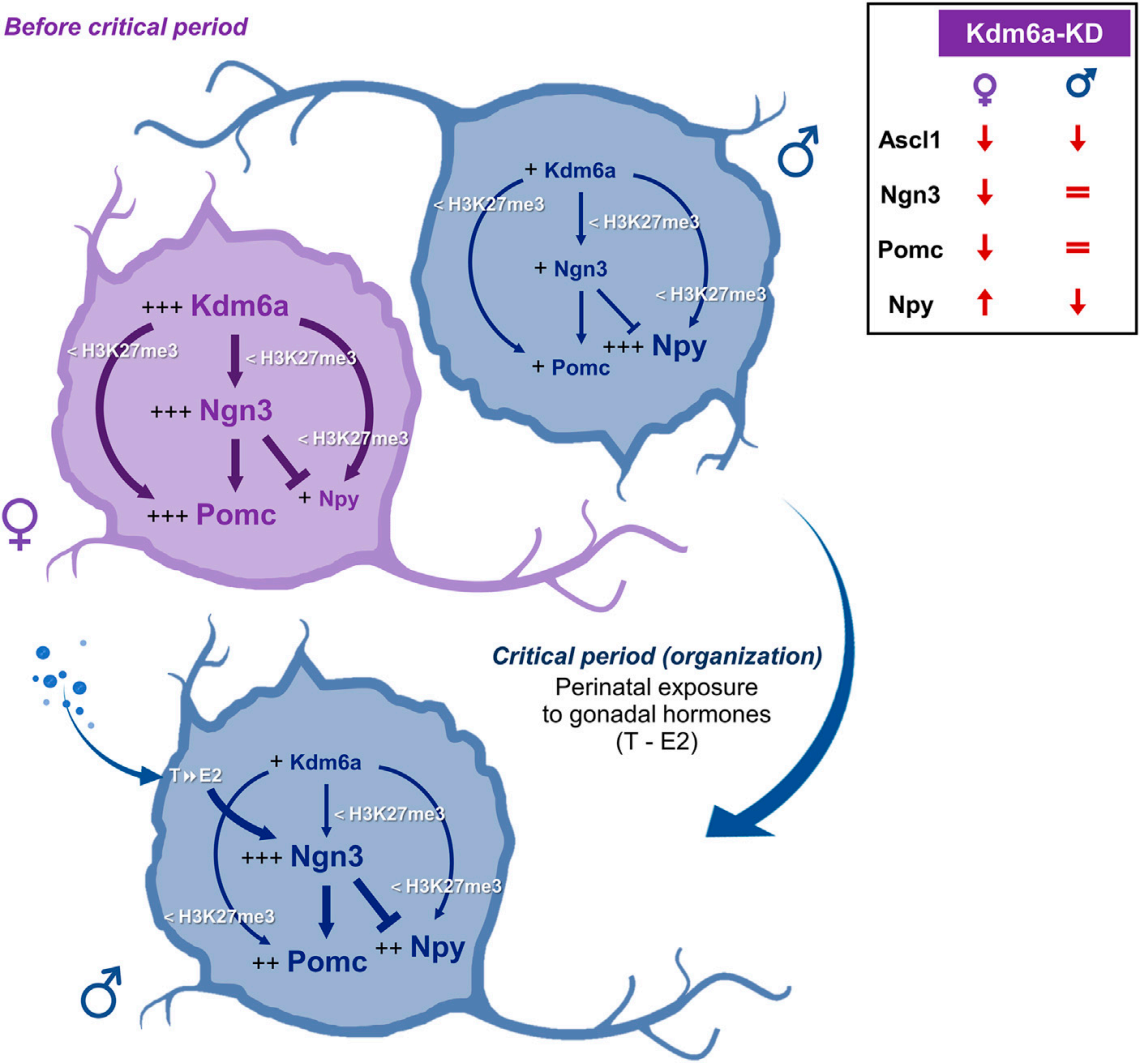
Hypothesis proposed to explain the sexually dimorphic mechanisms by which Kdm6a regulates the expression of Ngn3, Pomc, and Npy in the developing hypothalamus. Before the critical period of hormonal organization of the brain, female hypothalamic neurons show higher expression of the X-linked Kdm6a than male neurons (Cabrera Zapata et al., 2021). Higher Kdm6a levels in female neurons upregulate Ngn3 transcription through H3K27me3 demethylation. In turn, Ngn3 is a proneural transcription factor that has been reported to promote Pomc and repress Npy expression (Pelling et al., 2011) and could be mediating the higher Pomc and lower Npy levels observed in females when compared to males. In addition, Kdm6a promotes Ngn3, Pomc, and Npy transcription by removing repressive H3K27me3, with a stronger effect in females than in males, consistent with the increased levels of demethylase in females. Later, during the critical period, male but not female neurons are exposed to the effects of estradiol (E2) converted intraneuronally from gonadal testosterone (T). E2 organizational actions increase Ngn3 expression in males (Scerbo et al., 2014; Cisternas et al., 2020), which could lead to increased Ngn3-mediated effects on Pomc and Npy expression. Comparisons between sexes are indicated as follows: more plus signs (+) and larger font size indicate higher expression levels; thicker arrows/lines indicate a stronger effect. The top-right panel summarizes the Kdm6a knockdown (Kdm6a-KD) effects found on *Ascl1*, *Ngn3*, *Pomc*, and *Npy* expression.

Results regarding the neuropeptides Pomc and Npy show sex-dependent expression patterns at both mRNA and protein levels, with Pomc expressing more in female than in male hypothalamic neurons and Npy showing the opposite pattern of higher expression in male than in female neurons. siRNA targeting Kdm6a mRNA decreased Pomc expression only in female neurons without affecting its levels in male neurons. On the other hand, Kdm6a downregulation increased Npy expression in female neurons while decreasing it in males. Although at first glance these results may seem controversial, they can be easily explained considering the higher Kdm6a and Ngn3 expression levels in female than in male neurons and the opposite roles of Ngn3 in promoting Pomc and repressing Npy fates previously reported (Pelling et al., 2011; Anthwal et al., 2013). Consequently, in female neurons, the higher levels of Kdm6a upregulate Ngn3, which, in turn, increases Pomc and decreases Npy expression, while in male neurons, the lower levels of Kdm6a and Ngn3 may lead to favor Npy over Pomc expression (see Figure 6 for a summary and explanatory hypothesis of results).

Ngn3 is well known as a master regulator of pancreatic and gut endocrine cell fate specification (Gradwohl et al., 2000; Jenny et al., 2002; Gasa et al., 2004; Mellitzer et al., 2010). Although some studies have demonstrated cooperation between Ngn3, H3K27 Kdm6 demethylases, and other chromatin modifiers in the activation of downstream genes controlling pancreatic development (Fontcuberta-PiSunyer et al., 2018; Yu et al., 2018), no study to date has confirmed whether the promoter region of *Ngn3* is *per se* under regulation by H3K27 methylation marks, neither in pancreatic/enteroendocrine cells nor in neurons. As far as we are aware, our ChIP-qPCR data demonstrated for the first time that *Ngn3*, *Pomc*, and *Npy* promoters are occupied by the repressive H3K27me3 epigenetic motif and that their demethylation to upregulate their transcription depends specifically on Kdm6a among other H3K27 demethylases of the Kdm6 subfamily. Notably, we found significantly higher levels of H3K27me3 associated with the promoter regions of these genes in male than in female hypothalamic neurons, consistent with the higher expression and likely increased H3K27 demethylase activity of Kdm6a in females (Cabrera Zapata et al., 2021). These results together with results coming from mRNA and protein expression studies clearly show that Kdm6a controls the transcription of Ngn3, Pomc, and Npy in hypothalamic neurons in a sex-dependent manner, while strongly suggesting this regulation occurs through H3K27 demethylation without discarding other mechanisms also displayed by Kdm6a such as promotion of H3K4 methylation and/or H3K27 acetylation by association with COMPASS/COMPASS-like complexes and histone acetyltransferases, respectively (Cho et al., 2007; Tie et al., 2012; Wang et al., 2012; Wang et al., 2017).

Taken together, the results of the present study suggest a model whereby Kdm6a controls Pomc and Npy expression at the transcriptional and post-transcriptional levels in a sex-specific manner through H3K27 demethylation and regulation of the bHLH Ascl1/Ngn3 axis (Figure 6). On the one hand, female hypothalamic neurons, whose embryonic and perinatal development occurs in the absence of significant levels of gonadal hormones, show higher expression of the X-linked Kdm6a than male neurons (Cabrera Zapata et al., 2021 and Figure 1A). Higher Kdm6a levels in female neurons lead *via* H3K27 demethylation to an increase in Ngn3 levels, which, in turn, could be mediating the higher Pomc and lower Npy levels observed in females when compared to males through their opposite roles of promoting/repressing Pomc/Npy expression, respectively (Pelling et al., 2011). On the other hand, later in development during the so-called “critical period” of brain organization by gonadal hormones (E17-P10 in mice), developing testes start to secrete significant amounts of testosterone, leading to a perinatal exposure of male but not female neurons to the effects of 17β-estradiol (E2) converted intraneuronally from gonadal testosterone (Phoenix et al., 1959; Rhoda et al., 1984; O'Shaughnessy et al., 2006; McCarthy, 2008; Gagnidze et al., 2010). E2 organizational actions would upregulate Ngn3 expression in male hypothalamic neurons (Scerbo et al., 2014; Cisternas et al., 2020), which, subsequently, could lead to a potentiation of Ngn3-mediated effects, promoting Pomc and repressing Npy expression. In addition, Kdm6a promotes Ngn3, Pomc, and Npy transcription by removing repressive H3K27me3, with a stronger effect in females than in males, consistent with the increased levels of demethylase in females. Although gene specificity mechanisms by which X-linked Kdm6a regulates transcription of some autosomal genes (e.g., *Ngn3*, *Th*, *Pomc*, and *Npy*) but not others (e.g., *Gad1* and *Sf1*) in a sex-dependent manner are largely unknown, we hypothesize that this is a developmental stage-, locus-, and sex-specific mechanism involving Kdm6a interaction with other sexually dimorphic regulators such as sex hormone receptors, known to act as highly specific transcription factors (O'Lone et al., 2004; Kudwa et al., 2006; Cao and Patisaul, 2011; Cisternas et al., 2020; Gegenhuber et al., 2022). Interestingly, while we have previously demonstrated that the expression pattern of Kdm6a with higher mRNA levels in females than in males does not change under different conditions of gonadal hormone secretion/exposure, such as different developmental stages in the hypothalamus (before-E14-, during-P0-, and after-P60-critical period) or under E2 treatment in hypothalamic neurons *in vitro* (Cabrera Zapata et al., 2021), a recent study has shown that Kdm6a colocalizes with estrogen receptor alpha (ERα) on a subset of ERα target genes in an E2-dependent manner, cooperating with the receptor to upregulate the expression of these estrogen-responsive genes in a human breast cancer cell line (Xie et al., 2017). Further studies are needed to explore whether Kdm6a interacts with sex hormones and their receptors to mediate the sexually dimorphic neuronal differentiation and specification of neuronal subtypes in the hypothalamus.

The current study highlights the importance of an X-linked gene with a sexually dimorphic pattern of expression, the genome-wide epigenetic modifier Kdm6a, in the sex-specific control of transcription factors and neuropeptides pivotal in the development of the brain component of the feeding control circuit. We have demonstrated a critical requirement of Kdm6a for expression of the proneural bHLH Ascl1/Ngn3 transcriptional axis and the energy balance-related neuropeptides Pomc and Npy in a sex-dependent manner in hypothalamic neurons prior to the organizational actions of gonadal hormones on the developing brain, providing valuable new evidence for the impact of sex chromosomes on brain sexual differentiation beyond gonadal determination. Ngn3 is a developmental regulator of energy homeostasis systems in the hypothalamus, pancreas, and intestine, whereas Pomc+ and Npy+ hypothalamic neurons shape a central microcircuitry for the integration of peripheral signals providing feedback on energy status and for the control of feeding and energy expenditure; disruptions or deficits in any of these systems have been associated with hyperphagia, obesity, and obesity-related type 2 diabetes (Yaswen et al., 1999; Gradwohl et al., 2000; Anthwal et al., 2013; Bouret, 2017; Vohra et al., 2022). Notably, feeding and endocrine-related disorders such as feeding difficulties (some cases requiring long-term gastrostomy), gastrointestinal anomalies, tendency to overweight or obesity with age, hyperinsulinism, hypoglycemia, and diabetes insipidus are commonly reported in Kabuki syndrome specifically caused by KDM6A mutations, with male patients (carrying only one X chromosome and thus only one copy of KDM6A) tending to be more severely affected (Lederer et al., 2014; Banka et al., 2015; Gole et al., 2016; Wang et al., 2019; Faundes et al., 2021). Delving deeper into the genetic, epigenetic, and hormonal processes that regulate the development and function of the hypothalamus in each sex will lead to a better understanding of sexually dimorphic hypothalamic dysfunctions affecting energy homeostasis, allowing for more accurate treatments for epidemic diseases such as obesity in both men and women.

## Data Availability

The datasets generated and/or analyzed during the current study are available from the corresponding author on reasonable request.

## References

[B1] Alvarez-Bolado G. (2019). Development of neuroendocrine neurons in the mammalian hypothalamus. Cell Tissue Res. 375 (1), 23–39. 10.1007/s00441-018-2859-1 29869716

[B2] Anthwal N. Pelling M. Claxton S. Mellitzer G. Collin C. Kessaris N. (2013). Conditional deletion of neurogenin-3 using Nkx2.1iCre results in a mouse model for the central control of feeding, activity and obesity. Dis. Model. Mech. 6 (5), 1133–1145. 10.1242/dmm.011916 23649822 PMC3759333

[B3] Armoskus C. Moreira D. Bollinger K. Jimenez O. Taniguchi S. Tsai H. W. (2014). Identification of sexually dimorphic genes in the neonatal mouse cortex and hippocampus. Brain Res. 1562, 23–38. 10.1016/j.brainres.2014.03.017 24661915 PMC4058436

[B4] Aujla P. K. Naratadam G. T. Xu L. Raetzman L. T. (2013). Notch/Rbpjκ signaling regulates progenitor maintenance and differentiation of hypothalamic arcuate neurons. Development 140 (17), 3511–3521. 10.1242/dev.098681 23884446 PMC3742139

[B5] Baker N. E. Brown N. L. (2018). All in the family: Proneural bHLH genes and neuronal diversity. Development 145 (9), dev159426. 10.1242/dev.159426 29720483 PMC5992591

[B6] Balthasar N. Dalgaard L. T. Lee C. E. Yu J. Funahashi H. Williams T. (2005). Divergence of melanocortin pathways in the control of food intake and energy expenditure. Cell 123 (3), 493–505. 10.1016/j.cell.2005.08.035 16269339

[B7] Banka S. Lederer D. Benoit V. Jenkins E. Howard E. Bunstone S. (2015). Novel KDM6A (UTX) mutations and a clinical and molecular review of the X-linked Kabuki syndrome (KS2). Clin. Genet. 87 (3), 252–258. 10.1111/cge.12363 24527667

[B8] Bao A. M. Swaab D. F. (2010). Sex differences in the brain, behavior, and neuropsychiatric disorders. Neuroscientist 16 (5), 550–565. 10.1177/1073858410377005 20889965

[B9] Bedont J. L. Newman E. A. Blackshaw S. (2015). Patterning, specification, and differentiation in the developing hypothalamus. Wiley Interdiscip. Rev. Dev. Biol. 4 (5), 445–468. 10.1002/wdev.187 25820448 PMC5890958

[B10] Berletch J. B. Ma W. Yang F. Shendure J. Noble W. S. Disteche C. M. (2015). Escape from X inactivation varies in mouse tissues. PLoS Genet. 11 (3), e1005079. 10.1371/journal.pgen.1005079 25785854 PMC4364777

[B11] Berthois Y. Katzenellenbogen J. A. Katzenellenbogen B. S. (1986). Phenol red in tissue culture media is a weak estrogen: implications concerning the study of estrogen-responsive cells in culture. Proc. Natl. Acad. Sci. U. S. A. 83 (8), 2496–2500. 10.1073/pnas.83.8.2496 3458212 PMC323325

[B12] Bingham N. C. Anderson K. K. Reuter A. L. Stallings N. R. Parker K. L. (2008). Selective loss of leptin receptors in the ventromedial hypothalamic nucleus results in increased adiposity and a metabolic syndrome. Endocrinology 149 (5), 2138–2148. 10.1210/en.2007-1200 18258679 PMC2329259

[B13] Bogershausen N. Gatinois V. Riehmer V. Kayserili H. Becker J. Thoenes M. (2016). Mutation update for Kabuki syndrome genes KMT2D and KDM6A and further delineation of X-linked Kabuki syndrome subtype 2. Hum. Mutat. 37 (9), 847–864. 10.1002/humu.23026 27302555

[B14] Bouret S. G. (2017). “Development of hypothalamic circuits that control food intake and energy balance,” in Appetite and food intake: Central control. Editor Harris R. B. S. (Boca Raton (FL), 135–154. 28880512

[B15] Cabrera Zapata L. E. Cisternas C. D. Sosa C. Garcia-Segura L. M. Arevalo M. A. Cambiasso M. J. (2021). X-linked histone H3K27 demethylase Kdm6a regulates sexually dimorphic differentiation of hypothalamic neurons. Cell. Mol. Life Sci. 78 (21-22), 7043–7060. 10.1007/s00018-021-03945-0 34633482 PMC8558156

[B16] Cabrera Zapata L. E. Cambiasso M. J. Arevalo M. A. (2022). Epigenetic modifier kdm6a/utx controls the specification of hypothalamic neuronal subtypes in a sex-dependent manner. bioRxiv. [Preprint]. 10.1101/2022.07.25.501459 PMC9577230 36268511

[B17] Cao J. Patisaul H. B. (2011). Sexually dimorphic expression of hypothalamic estrogen receptors alpha and beta and Kiss1 in neonatal male and female rats. J. Comp. Neurol. 519 (15), 2954–2977. 10.1002/cne.22648 21484804 PMC3874381

[B18] Chen X. Xiao Z. Cai Y. Huang L. Chen C. (2022). Hypothalamic mechanisms of obesity-associated disturbance of hypothalamic-pituitary-ovarian axis. Trends Endocrinol. Metab. 33 (3), 206–217. 10.1016/j.tem.2021.12.004 35063326

[B19] Cho Y. W. Hong T. Hong S. Guo H. Yu H. Kim D. (2007). PTIP associates with MLL3- and MLL4-containing histone H3 lysine 4 methyltransferase complex. J. Biol. Chem. 282 (28), 20395–20406. 10.1074/jbc.M701574200 17500065 PMC2729684

[B20] Cisternas C. D. Tome K. Caeiro X. E. Dadam F. M. Garcia-Segura L. M. Cambiasso M. J. (2015). Sex chromosome complement determines sex differences in aromatase expression and regulation in the stria terminalis and anterior amygdala of the developing mouse brain. Mol. Cell. Endocrinol. 414, 99–110. 10.1016/j.mce.2015.07.027 26231585

[B21] Cisternas C. D. Cabrera Zapata L. E. Mir F. R. Scerbo M. J. Arevalo M. A. Garcia-Segura L. M. (2020). Estradiol-dependent axogenesis and Ngn3 expression are determined by XY sex chromosome complement in hypothalamic neurons. Sci. Rep. 10 (1), 8223. 10.1038/s41598-020-65183-x 32427857 PMC7237695

[B22] Coll A. P. Yeo G. S. (2013). The hypothalamus and metabolism: integrating signals to control energy and glucose homeostasis. Curr. Opin. Pharmacol. 13 (6), 970–976. 10.1016/j.coph.2013.09.010 24075719

[B23] Davis E. J. Broestl L. Abdulai-Saiku S. Worden K. Bonham L. W. Minones-Moyano E. (2020). A second X chromosome contributes to resilience in a mouse model of Alzheimer's disease. Sci. Transl. Med. 12 (558), eaaz5677. 10.1126/scitranslmed.aaz5677 32848093 PMC8409261

[B24] Dou Z. Son J. E. Hui C. C. (2021). Irx3 and Irx5 - novel regulatory factors of postnatal hypothalamic neurogenesis. Front. Neurosci. 15, 763856. 10.3389/fnins.2021.763856 34795556 PMC8593166

[B25] Farooqi I. S. (2022). Monogenic obesity syndromes provide insights into the hypothalamic regulation of appetite and associated behaviors. Biol. Psychiatry 91, 856–859. 10.1016/j.biopsych.2022.01.018 35369984

[B26] Faundes V. Goh S. Akilapa R. Bezuidenhout H. Bjornsson H. T. Bradley L. (2021). Clinical delineation, sex differences, and genotype-phenotype correlation in pathogenic KDM6A variants causing X-linked Kabuki syndrome type 2. Genet. Med. 23 (7), 1202–1210. 10.1038/s41436-021-01119-8 33674768 PMC8257478

[B27] Fontcuberta-PiSunyer M. Cervantes S. Miquel E. Mora-Castilla S. Laurent L. C. Raya A. (2018). Modulation of the endocrine transcriptional program by targeting histone modifiers of the H3K27me3 mark. Biochim. Biophys. Acta. Gene Regul. Mech. 1861 (5), 473–480. 10.1016/j.bbagrm.2018.03.003 29530603

[B28] Gagnidze K. Pfaff D. W. Mong J. A. (2010). Gene expression in neuroendocrine cells during the critical period for sexual differentiation of the brain. Prog. Brain Res. 186, 97–111. 10.1016/B978-0-444-53630-3.00007-5 21094888

[B29] Gasa R. Mrejen C. Leachman N. Otten M. Barnes M. Wang J. (2004). Proendocrine genes coordinate the pancreatic islet differentiation program *in vitro* . Proc. Natl. Acad. Sci. U. S. A. 101 (36), 13245–13250. 10.1073/pnas.0405301101 15340143 PMC516555

[B30] Gautron L. Elmquist J. K. Williams K. W. (2015). Neural control of energy balance: translating circuits to therapies. Cell 161 (1), 133–145. 10.1016/j.cell.2015.02.023 25815991 PMC4392840

[B31] Gegenhuber B. Wu M. V. Bronstein R. Tollkuhn J. (2022). Gene regulation by gonadal hormone receptors underlies brain sex differences. Nature 606 (7912), 153–159. 10.1038/s41586-022-04686-1 35508660 PMC9159952

[B32] Global B. M. I. M. C. Di Angelantonio E. Bhupathiraju Sh N. Wormser D. Gao P. Kaptoge S. (2016). Body-mass index and all-cause mortality: individual-participant-data meta-analysis of 239 prospective studies in four continents. Lancet 388 (10046), 776–786. 10.1016/s0140-6736(16)30175-1 27423262 PMC4995441

[B33] Gole H. Chuk R. Coman D. (2016). Persistent hyperinsulinism in Kabuki syndrome 2: Case report and literature review. Clin. Pract. 6 (3), 848. 10.4081/cp.2016.848 27777708 PMC5067400

[B34] Gradwohl G. Dierich A. LeMeur M. Guillemot F. (2000). neurogenin3 is required for the development of the four endocrine cell lineages of the pancreas. Proc. Natl. Acad. Sci. U. S. A. 97 (4), 1607–1611. 10.1073/pnas.97.4.1607 10677506 PMC26482

[B35] Greenfield A. Carrel L. Pennisi D. Philippe C. Quaderi N. Siggers P. (1998). The UTX gene escapes X inactivation in mice and humans. Hum. Mol. Genet. 7 (4), 737–742. 10.1093/hmg/7.4.737 9499428

[B36] Gropp E. Shanabrough M. Borok E. Xu A. W. Janoschek R. Buch T. (2005). Agouti-related peptide-expressing neurons are mandatory for feeding. Nat. Neurosci. 8 (10), 1289–1291. 10.1038/nn1548 16158063

[B37] Hales C. M. Carroll M. D. Fryar C. D. Ogden C. L. (2020). Prevalence of obesity and severe obesity among adults: United States, 2017-2018. NCHS Data Brief. (360), 1–8. 32487284

[B38] Hong S. Cho Y. W. Yu L. R. Yu H. Veenstra T. D. Ge K. (2007). Identification of JmjC domain-containing UTX and JMJD3 as histone H3 lysine 27 demethylases. Proc. Natl. Acad. Sci. U. S. A. 104 (47), 18439–18444. 10.1073/pnas.0707292104 18003914 PMC2141795

[B39] Hourai A. Miyata S. (2013). Neurogenesis in the circumventricular organs of adult mouse brains. J. Neurosci. Res. 91 (6), 757–770. 10.1002/jnr.23206 23526379

[B40] Huang C. Chan J. A. Schuurmans C. (2014). Proneural bHLH genes in development and disease. Curr. Top. Dev. Biol. 110, 75–127. 10.1016/B978-0-12-405943-6.00002-6 25248474

[B41] Jenny M. Uhl C. Roche C. Duluc I. Guillermin V. Guillemot F. (2002). Neurogenin3 is differentially required for endocrine cell fate specification in the intestinal and gastric epithelium. EMBO J. 21 (23), 6338–6347. 10.1093/emboj/cdf649 12456641 PMC136953

[B42] Khanna D. Khanna S. Khanna P. Kahar P. Patel B. M. (2022). Obesity: A chronic low-grade inflammation and its markers. Cureus 14 (2), e22711. 10.7759/cureus.22711 35386146 PMC8967417

[B43] Kokoeva M. V. Yin H. Flier J. S. (2005). Neurogenesis in the hypothalamus of adult mice: Potential role in energy balance. Science 310 (5748), 679–683. 10.1126/science.1115360 16254185

[B44] Kokoeva M. V. Yin H. Flier J. S. (2007). Evidence for constitutive neural cell proliferation in the adult murine hypothalamus. J. Comp. Neurol. 505 (2), 209–220. 10.1002/cne.21492 17853440

[B45] Krause W. C. Ingraham H. A. (2017). Origins and functions of the ventrolateral VMH: A complex neuronal cluster orchestrating sex differences in metabolism and behavior. Adv. Exp. Med. Biol. 1043, 199–213. 10.1007/978-3-319-70178-3_10 29224096 PMC5839321

[B46] Kudwa A. E. Michopoulos V. Gatewood J. D. Rissman E. F. (2006). Roles of estrogen receptors alpha and beta in differentiation of mouse sexual behavior. Neuroscience 138 (3), 921–928. 10.1016/j.neuroscience.2005.10.018 16338079

[B47] Lederer D. Shears D. Benoit V. Verellen-Dumoulin C. Maystadt I. (2014). A three generation X-linked family with Kabuki syndrome phenotype and a frameshift mutation in KDM6A. Am. J. Med. Genet. A 164A (5), 1289–1292. 10.1002/ajmg.a.36442 24664873

[B48] Lei X. Jiao J. (2018). UTX affects neural stem cell proliferation and differentiation through PTEN signaling. Stem Cell Rep. 10 (4), 1193–1207. 10.1016/j.stemcr.2018.02.008 PMC5998300 29551674

[B49] Leibowitz S. F. (1991). Brain neuropeptide Y: an integrator of endocrine, metabolic and behavioral processes. Brain Res. Bull. 27 (3-4), 333–337. 10.1016/0361-9230(91)90121-y 1959027

[B50] Lustig R. H. Collier D. Kassotis C. Roepke T. A. Ji Kim M. Blanc E. (2022). Obesity I: Overview and molecular and biochemical mechanisms. Biochem. Pharmacol. 199, 115012. 10.1016/j.bcp.2022.115012 35393120 PMC9050949

[B51] Martin-Rodriguez E. Guillen-Grima F. Marti A. Brugos-Larumbe A. (2015). Comorbidity associated with obesity in a large population: The APNA study. Obes. Res. Clin. Pract. 9 (5), 435–447. 10.1016/j.orcp.2015.04.003 25979684

[B52] McCarthy M. M. (2008). Estradiol and the developing brain. Physiol. Rev. 88 (1), 91–124. 10.1152/physrev.00010.2007 18195084 PMC2754262

[B53] McEwen B. S. Davis P. G. Parsons B. Pfaff D. W. (1979). The brain as a target for steroid hormone action. Annu. Rev. Neurosci. 2, 65–112. 10.1146/annurev.ne.02.030179.000433 395885

[B54] McEwen B. S. (1981). Neural gonadal steroid actions. Science 211 (4488), 1303–1311. 10.1126/science.6259728 6259728

[B55] McNay D. E. Pelling M. Claxton S. Guillemot F. Ang S. L. (2006). Mash1 is required for generic and subtype differentiation of hypothalamic neuroendocrine cells. Mol. Endocrinol. 20 (7), 1623–1632. 10.1210/me.2005-0518 16469766

[B56] Mellitzer G. Beucher A. Lobstein V. Michel P. Robine S. Kedinger M. (2010). Loss of enteroendocrine cells in mice alters lipid absorption and glucose homeostasis and impairs postnatal survival. J. Clin. Invest. 120 (5), 1708–1721. 10.1172/JCI40794 20364088 PMC2860910

[B57] Miyake N. Mizuno S. Okamoto N. Ohashi H. Shiina M. Ogata K. (2013). KDM6A point mutations cause Kabuki syndrome. Hum. Mutat. 34 (1), 108–110. 10.1002/humu.22229 23076834

[B58] Morton G. J. Cummings D. E. Baskin D. G. Barsh G. S. Schwartz M. W. (2006). Central nervous system control of food intake and body weight. Nature 443 (7109), 289–295. 10.1038/nature05026 16988703

[B59] Mountjoy K. G. (2015). Pro-Opiomelanocortin (POMC) neurones, POMC-derived peptides, melanocortin receptors and obesity: How understanding of this system has changed over the last decade. J. Neuroendocrinol. 27 (6), 406–418. 10.1111/jne.12285 25872650

[B60] O'Lone R. Frith M. C. Karlsson E. K. Hansen U. (2004). Genomic targets of nuclear estrogen receptors. Mol. Endocrinol. 18 (8), 1859–1875. 10.1210/me.2003-0044 15031323

[B61] O'Shaughnessy P. J. Baker P. J. Johnston H. (2006). The foetal Leydig cell-- differentiation, function and regulation. Int. J. Androl. 29 (1), 90–95. discussion 105-108. 10.1111/j.1365-2605.2005.00555.x 16466528

[B62] Pelling M. Anthwal N. McNay D. Gradwohl G. Leiter A. B. Guillemot F. (2011). Differential requirements for neurogenin 3 in the development of POMC and NPY neurons in the hypothalamus. Dev. Biol. 349 (2), 406–416. 10.1016/j.ydbio.2010.11.007 21074524

[B63] Pfaff D. W. Christen Y. (2013). Multiple origins of sex differences in brain : neuroendocrine functions and their pathologies. New York: Springer.

[B64] Pfaffl M. W. Tichopad A. Prgomet C. Neuvians T. P. (2004). Determination of stable housekeeping genes, differentially regulated target genes and sample integrity: BestKeeper--Excel-based tool using pair-wise correlations. Biotechnol. Lett. 26 (6), 509–515. 10.1023/b:bile.0000019559.84305.47 15127793

[B65] Phoenix C. H. Goy R. W. Gerall A. A. Young W. C. (1959). Organizing action of prenatally administered testosterone propionate on the tissues mediating mating behavior in the female Guinea pig. Endocrinology 65, 369–382. 10.1210/endo-65-3-369 14432658

[B66] Regensteiner J. G. Reusch J. E. B. (2022). Sex differences in cardiovascular consequences of hypertension, obesity, and diabetes: JACC focus seminar 4/7. J. Am. Coll. Cardiol. 79 (15), 1492–1505. 10.1016/j.jacc.2022.02.010 35422246 PMC9503760

[B67] Rhoda J. Corbier P. Roffi J. (1984). Gonadal steroid concentrations in serum and hypothalamus of the rat at birth: aromatization of testosterone to 17 beta-estradiol. Endocrinology 114 (5), 1754–1760. 10.1210/endo-114-5-1754 6714163

[B68] Romanov R. A. Tretiakov E. O. Kastriti M. E. Zupancic M. Haring M. Korchynska S. (2020). Molecular design of hypothalamus development. Nature 582 (7811), 246–252. 10.1038/s41586-020-2266-0 32499648 PMC7292733

[B69] Ruiz-Palmero I. Ortiz-Rodriguez A. Melcangi R. C. Caruso D. Garcia-Segura L. M. Rune G. M. (2016). Oestradiol synthesized by female neurons generates sex differences in neuritogenesis. Sci. Rep. 6, 31891. 10.1038/srep31891 27553191 PMC4995407

[B70] Scerbo M. J. Freire-Regatillo A. Cisternas C. D. Brunotto M. Arevalo M. A. Garcia-Segura L. M. (2014). Neurogenin 3 mediates sex chromosome effects on the generation of sex differences in hypothalamic neuronal development. Front. Cell. Neurosci. 8, 188. 10.3389/fncel.2014.00188 25071448 PMC4086225

[B71] Seidell J. C. (2005). Epidemiology of obesity. Semin. Vasc. Med. 5 (1), 3–14. 10.1055/s-2005-871737 15968575

[B72] Shan Y. Zhang Y. Zhao Y. Wang T. Zhang J. Yao J. (2020). JMJD3 and UTX determine fidelity and lineage specification of human neural progenitor cells. Nat. Commun. 11 (1), 382. 10.1038/s41467-019-14028-x 31959746 PMC6971254

[B73] Shpargel K. B. Sengoku T. Yokoyama S. Magnuson T. (2012). UTX and UTY demonstrate histone demethylase-independent function in mouse embryonic development. PLoS Genet. 8 (9), e1002964. 10.1371/journal.pgen.1002964 23028370 PMC3459986

[B74] Son J. E. Dou Z. Kim K. H. Wanggou S. Cha V. S. B. Mo R. (2021). Irx3 and Irx5 in Ins2-Cre(+) cells regulate hypothalamic postnatal neurogenesis and leptin response. Nat. Metab. 3 (5), 701–713. 10.1038/s42255-021-00382-y 33859429

[B75] Subhramanyam C. S. Cao Q. Wang C. Heng Z. S. L. Zhou Z. Hu Q. (2020). Role of PIWI-like 4 in modulating neuronal differentiation from human embryonal carcinoma cells. RNA Biol. 17, 1613–1624. 10.1080/15476286.2020.1757896 32372724 PMC7567516

[B76] Sueda R. Imayoshi I. Harima Y. Kageyama R. (2019). High Hes1 expression and resultant Ascl1 suppression regulate quiescent vs. active neural stem cells in the adult mouse brain. Genes Dev. 33 (9-10), 511–523. 10.1101/gad.323196.118 30862661 PMC6499325

[B77] Tang G. B. Zeng Y. Q. Liu P. P. Mi T. W. Zhang S. F. Dai S. K. (2017). The histone H3K27 demethylase UTX regulates synaptic plasticity and cognitive behaviors in mice. Front. Mol. Neurosci. 10, 267. 10.3389/fnmol.2017.00267 28970783 PMC5609596

[B78] Tang Q. Y. Zhang S. F. Dai S. K. Liu C. Wang Y. Y. Du H. Z. (2020). UTX regulates human neural differentiation and dendritic morphology by resolving bivalent promoters. Stem Cell Rep. 15, 439–453. 10.1016/j.stemcr.2020.06.015 PMC7419705 32679064

[B79] Teufel F. Seiglie J. A. Geldsetzer P. Theilmann M. Marcus M. E. Ebert C. (2021). Body-mass index and diabetes risk in 57 low-income and middle-income countries: a cross-sectional study of nationally representative, individual-level data in 685 616 adults. Lancet 398 (10296), 238–248. 10.1016/S0140-6736(21)00844-8 34274065 PMC8336025

[B80] Tie F. Banerjee R. Conrad P. A. Scacheri P. C. Harte P. J. (2012). Histone demethylase UTX and chromatin remodeler BRM bind directly to CBP and modulate acetylation of histone H3 lysine 27. Mol. Cell. Biol. 32 (12), 2323–2334. 10.1128/MCB.06392-11 22493065 PMC3372260

[B81] Timper K. Bruning J. C. (2017). Hypothalamic circuits regulating appetite and energy homeostasis: Pathways to obesity. Dis. Model. Mech. 10 (6), 679–689. 10.1242/dmm.026609 28592656 PMC5483000

[B82] Tran N. Broun A. Ge K. (2020). Lysine demethylase KDM6A in differentiation, development, and cancer. Mol. Cell. Biol. 40 (20), 003411–e420. 10.1128/MCB.00341-20 PMC7523656 32817139

[B83] Tsao C. W. Aday A. W. Almarzooq Z. I. Alonso A. Beaton A. Z. Bittencourt M. S. (2022). Heart disease and stroke statistics-2022 update: A report from the American heart association. Circulation 145 (8), e153–e639. 10.1161/CIR.0000000000001052 35078371

[B84] Tukiainen T. Villani A. C. Yen A. Rivas M. A. Marshall J. L. Satija R. (2017). Landscape of X chromosome inactivation across human tissues. Nature 550 (7675), 244–248. 10.1038/nature24265 29022598 PMC5685192

[B85] Uwaifo G. I. (2021). The human hypothalamus - anatomy, dysfunction and disease management. Cham: Humana Press, Springer Nature Switzerland AG.

[B86] Van Laarhoven P. M. Neitzel L. R. Quintana A. M. Geiger E. A. Zackai E. H. Clouthier D. E. (2015). Kabuki syndrome genes KMT2D and KDM6A: functional analyses demonstrate critical roles in craniofacial, heart and brain development. Hum. Mol. Genet. 24 (15), 4443–4453. 10.1093/hmg/ddv180 25972376 PMC4492403

[B87] Vasquez-Avila K. Pacheco-Barrios K. de Melo P. S. Fregni F. (2021). Addressing the critical role of gender identity and sex in the planning, analysis, and conduct of clinical trials. Princ. Pract. Clin. Res. 7 (2), 59–62. 10.21801/ppcrj.2021.72.7 34532571 PMC8443128

[B88] Vohra M. S. Benchoula K. Serpell C. J. Hwa W. E. (2022). AgRP/NPY and POMC neurons in the arcuate nucleus and their potential role in treatment of obesity. Eur. J. Pharmacol. 915, 174611. 10.1016/j.ejphar.2021.174611 34798121

[B89] Wang C. Lee J. E. Cho Y. W. Xiao Y. Jin Q. Liu C. (2012). UTX regulates mesoderm differentiation of embryonic stem cells independent of H3K27 demethylase activity. Proc. Natl. Acad. Sci. U. S. A. 109 (38), 15324–15329. 10.1073/pnas.1204166109 22949634 PMC3458330

[B90] Wang S. P. Tang Z. Chen C. W. Shimada M. Koche R. P. Wang L. H. (2017). A UTX-MLL4-p300 transcriptional regulatory network coordinately shapes active enhancer landscapes for eliciting transcription. Mol. Cell 67 (2), 308–321. 10.1016/j.molcel.2017.06.028 28732206 PMC5574165

[B91] Wang Y. R. Xu N. X. Wang J. Wang X. M. (2019). Kabuki syndrome: review of the clinical features, diagnosis and epigenetic mechanisms. World J. Pediatr. 15 (6), 528–535. 10.1007/s12519-019-00309-4 31587141

[B92] Xie G. Liu X. Zhang Y. Li W. Liu S. Chen Z. (2017). UTX promotes hormonally responsive breast carcinogenesis through feed-forward transcription regulation with estrogen receptor. Oncogene 36 (39), 5497–5511. 10.1038/onc.2017.157 28534508

[B93] Xu J. Deng X. Watkins R. Disteche C. M. (2008). Sex-specific differences in expression of histone demethylases Utx and Uty in mouse brain and neurons. J. Neurosci. 28 (17), 4521–4527. 10.1523/JNEUROSCI.5382-07.2008 18434530 PMC2643472

[B94] Yang X. Xu B. Mulvey B. Evans M. Jordan S. Wang Y. D. (2019). Differentiation of human pluripotent stem cells into neurons or cortical organoids requires transcriptional co-regulation by UTX and 53BP1. Nat. Neurosci. 22 (3), 362–373. 10.1038/s41593-018-0328-5 30718900 PMC6511450

[B95] Yaswen L. Diehl N. Brennan M. B. Hochgeschwender U. (1999). Obesity in the mouse model of pro-opiomelanocortin deficiency responds to peripheral melanocortin. Nat. Med. 5 (9), 1066–1070. 10.1038/12506 10470087

[B96] Yoo S. Blackshaw S. (2018). Regulation and function of neurogenesis in the adult mammalian hypothalamus. Prog. Neurobiol. 170, 53–66. 10.1016/j.pneurobio.2018.04.001 29631023 PMC6173995

[B97] Yu X. X. Qiu W. L. Yang L. Li L. C. Zhang Y. W. Xu C. R. (2018). Dynamics of chromatin marks and the role of JMJD3 during pancreatic endocrine cell fate commitment. Development 145 (6), dev163162. 10.1242/dev.163162 29559448

